# Comparison of Two Different Doses of Prophylactic Intravenous Magnesium Sulphate for the Prevention of Shivering in Patients Undergoing Procedures Under Spinal Anesthesia

**DOI:** 10.7759/cureus.99905

**Published:** 2025-12-23

**Authors:** Chrish Lenil, Akash Yadhu, Rangapriya Aravindan, Shravya R

**Affiliations:** 1 Anesthesiology, Sree Balaji Medical College and Hospital, Chennai, IND

**Keywords:** hypothermia, magnesium sulfate, perioperative care, shivering, spinal anesthesia

## Abstract

Introduction: Shivering is a common and distressing complication of spinal anesthesia associated with increased metabolic demand, hemodynamic fluctuations, and impaired monitoring. Magnesium sulfate (MgSO_4_), an N-methyl-D-aspartate (NMDA) receptor antagonist, has been investigated for its thermoregulatory benefits. This study compared two prophylactic intravenous (IV) doses of magnesium sulfate (25 mg/kg vs 50 mg/kg) for the prevention of shivering in patients undergoing surgeries under spinal anesthesia.

Materials and methods: A prospective observational study was conducted at a tertiary care center over one year among 88 adult patients undergoing elective procedures under spinal anesthesia. Participants were allocated into two groups: Group A (50 mg/kg IV magnesium sulfate bolus) and Group B (25 mg/kg IV magnesium sulfate bolus). Hemodynamic parameters, temperature, and shivering grades were monitored intraoperatively and postoperatively. Shivering was graded on a 0-4 scale. Data were analyzed using IBM SPSS Statistics version 20 (IBM Corp., Armonk, USA); p < 0.05 was considered statistically significant.

Results: Demographic characteristics were comparable between groups. No significant differences were observed in heart rate, systolic/diastolic blood pressure, peripheral oxygen saturation (SpO_2_), or temperature at any time point (p > 0.05). Overall, 93.2% of patients did not develop shivering: 42/44 (95.5%) in Group A and 40/44 (90.9%) in Group B, with no statistically significant difference in shivering incidence or grade (p > 0.97). Shivering onset was clinically delayed in Group A, though without statistical significance.

Conclusion: Both 25 mg/kg and 50 mg/kg prophylactic IV magnesium sulfate effectively prevented shivering after spinal anesthesia without significant hemodynamic instability. The lower dose (25 mg/kg) demonstrated equivalent efficacy and safety, supporting its use as a preferable dose for routine prophylaxis.

## Introduction

Shivering is a frequent sequela of neuraxial anesthesia. This involuntary, rhythmic muscular activity serves to elevate body temperature via augmented metabolic heat production. While hypothermia can trigger shivering, it can also manifest in normothermic individuals [1].

Regional anesthesia impairs both central and peripheral thermoregulatory mechanisms. In individuals experiencing significant hypothermia, involuntary muscle contractions may manifest, often causing distress to patients and clinical staff alike [2].

Shivering can impede the accurate observation of blood pressure, peripheral oxygen saturation (SpO_2_), and cardiac rhythm. This physiological response is associated with substantial sympathetic nervous system activation. In patients exhibiting compromised cardiovascular and respiratory function, the associated elevation in metabolic rate, often accompanied by a quadrupling of oxygen consumption, coupled with lactic acid accumulation and amplified carbon dioxide production, may precipitate complications [3].

Thermoregulatory shivering represents the dominant form of this physiological response, occurring concurrently with peripheral vasoconstriction as a consequence of decreased body temperature [4]. In contrast, non-thermoregulatory shivering constitutes a minority of shivering events, accounting for only approximately 15% [2], and is correlated with peripheral vasodilation, along with nociception and psychological strain. Shivering manifests with two separate muscle activation modes as a result of disinhibited spinal modulation: a rapid, oscillatory pattern at a frequency of 5-7 Hz (clonic), and a slower, rhythmic pattern with a repetition rate of 4-8 cycles per minute (tonic), which resembles shivering induced by thermoregulation [5].

Although the point at which shivering begins in individuals not under anesthesia is 35.5°C, perioperative hypothermia is clinically identified as a core body temperature less than 36°C [6]. Anesthetic agents alter thermoregulation by elevating both the upper and lower temperature boundaries of the usual hypothalamic control range [7]. Intraoperative shivering can be especially detrimental in patients with elevated metabolic rates and limited cardiopulmonary function, as it is associated with increased oxygen consumption, carbon dioxide production, lactic acidosis, elevated intraocular pressure, and increased intracranial pressure. Furthermore, shivering complicates the monitoring of vital signs during surgery and reduces mixed venous oxygen saturation. Postoperative shivering can prolong hospitalization, intensify pain at the surgical site, and negatively affect the healing process [8].

While pharmacological interventions like meperidine, 5-hydroxytryptamine receptor antagonists, and dexmedetomidine can mitigate shivering, concerns regarding their safety profiles and financial implications exist [9]. Perioperative magnesium administration may reduce the need for opioids, neuromuscular blockers, and anesthetic agents [10,11]. Although a previous meta-analysis suggested that magnesium decreased shivering, other meta-analyses have yielded inconsistent findings. This discrepancy may arise from the limited number of studies included that evaluated shivering as a primary endpoint, with numerous studies assessing shivering as a secondary outcome or adverse effect being excluded. Consequently, the efficacy of prophylactic magnesium sulfate (MgSO_4_) at varying dosages for managing perioperative shivering following spinal anesthesia remains uncertain. Given the increasing prevalence of intravenous (IV) magnesium administration, further investigation is warranted [12].

Magnesium sulfate, acting as an N-methyl-D-aspartate (NMDA) receptor antagonist, reduces the shivering threshold when employed for postoperative shivering management [13]. Its safety is well-established, exhibiting a benign side effect profile and negligible influence on hemodynamic parameters when administered intrathecally [14]. While IV magnesium sulfate has been extensively investigated for shivering prevention, intrathecal magnesium sulfate [15] has received limited experimental attention. Therefore, this study was conducted to compare two routinely used prophylactic IV magnesium sulfate doses (25 mg/kg and 50 mg/kg) with respect to the primary outcomes of shivering incidence and shivering grade, as well as secondary outcomes of hemodynamic parameters and perioperative temperature changes in patients undergoing procedures under spinal anesthesia.

## Materials and methods

Study design, setting, and duration

This was a prospective comparative observational study conducted at the Department of Anesthesiology, Sree Balaji Medical College and Hospital, Chennai, India, over a one-year period from December 2023 to March 2025.

Study population (inclusion and exclusion criteria)

The study population included patients who underwent elective surgery under spinal anesthesia. Inclusion criteria comprised patients of both sexes aged 18-60 years with American Society of Anesthesiologists (ASA) physical status class I and II [16]. Exclusion criteria included hemodynamic instability, cardiopulmonary, renal, and liver diseases, thyroid disorders, known psychiatric disorders, coagulation abnormalities, cerebrovascular insufficiency, body mass index (BMI) > 35 kg/m^2^, patients on vasoactive medications or beta blockers, patients allergic to study drugs, those who received blood transfusions or more than 2000 mL of fluid intraoperatively, and pregnant women.

Sampling method

Participants were enrolled using consecutive sampling, whereby all eligible patients undergoing spinal anesthesia during the study period were included based on predefined criteria.

Sample size

The sample size was calculated using the formula for comparing two means. The standard deviation (σ = 20) and expected mean difference (µ1-µ2 = 5) were adapted from variability reported in comparable anesthesiology research by Darlong et al. [17].

Accordingly,

n = 2[(a + b)²σ²]/(µ1 - µ2)

n = 2[(1.96 + 0.842)² × 20²]/5²

n ≃ 44

where n represents the sample size in each of the groups; µ1 is the population mean in treatment Group A; µ2 is the population mean in treatment Group B; µ1 - µ2 is the difference the investigator wishes to detect; σ² denotes population variance; a is the conventional multiplier for alpha = 1.96; and b is the conventional multiplier for power = 0.842.

Thus, a total of 88 participants (44 per group) were included in the study.

No attrition occurred during the study period. All 88 enrolled participants completed intraoperative and postoperative assessments and were included in the final analysis.

Procedure

Following documented informed consent, data were collected using a pre-designed and pre-tested questionnaire that captured demographic details (age, sex, ASA classification), baseline clinical parameters (heart rate, blood pressure, SpO_2_, axillary temperature), surgical characteristics, and intraoperative monitoring variables.

Group A patients were given an IV bolus of magnesium sulfate at a dose of 50 mg/kg, while Group B patients received an IV bolus of magnesium sulfate at a dose of 25 mg/kg.

If shivering persisted, an additional 25 mg/kg IV magnesium sulfate bolus was used as rescue therapy. This approach was selected to ensure consistency of pharmacological exposure within the study population, to avoid confounding effects from the introduction of other anti-shivering agents (e.g., meperidine or tramadol), and because magnesium sulfate has a favorable safety profile, minimal hemodynamic impact, and proven efficacy generally.

In accordance with routine departmental practice, patients received either 25 mg/kg or 50 mg/kg of IV magnesium sulfate as part of the standard prophylactic protocol adopted by different consultant anesthesiologists. Magnesium sulfate was administered immediately before initiation of spinal anesthesia, as per routine departmental practice. The research team did not influence dose selection. Participants were observed in whichever dosing regimen they received during routine care, thereby minimizing investigator-driven allocation and maintaining an observational design.

Following IV access, all participants received 500 cc of room-temperature lactated Ringer's solution. No pre-medications were administered within the operating room. The ambient temperature was maintained between 22°C and 24°C, and no active warming measures (e.g., forced-air warming) were used. Spinal anesthesia was induced using a 24/25-gauge spinal needle (Polymed, India) to inject 0.5% hyperbaric bupivacaine (dose adjusted according to individual needs) at the L3/4 or L4/5 intervertebral spaces, with the patient in a seated position. Supplemental oxygen was administered via a Hudson oxygen mask (4 L/min) (Teleflex Medical, Research Triangle Park, USA) as needed.

Each case was continuously monitored throughout the surgical procedure and covered by a single layer of surgical drapes. Standard monitoring equipment (Philips Healthcare, Amsterdam, The Netherlands) was utilized to assess temperature, respiratory rate (cycles/min), heart rate, mean arterial pressure, and peripheral oxygen saturation. These physiological parameters were recorded prior to surgery, post-anesthesia induction, every 20 minutes during the surgical procedure, and every 20 minutes postoperatively until the sensory blockade had regressed by two dermatomal segments.

Temperature monitoring was performed using a calibrated digital thermometer (Omron Healthcare Co. Ltd., Kyoto, Japan). Axillary temperature was recorded as the surrogate for core temperature, as per routine clinical practice at the institution. Skin temperature was simultaneously measured on the lateral forearm using the same device in surface-temperature mode, ensuring uniformity across participants. The temperature differential (ΔT) was calculated as the numerical difference between the axillary (core surrogate) temperature and the peripheral skin temperature. Hypothermia was defined as an axillary temperature below 36°C. Shivering was assessed using the Crossley and Mahajan Shivering Scale: 0 = no shivering, 1 = piloerection or peripheral cyanosis without visible muscle activity, 2 = visible muscular activity confined to one muscle group, 3 = visible muscular activity in more than one muscle group, 4 = gross muscular activity involving the whole body [18,19]. This assessment was performed following the subarachnoid injection until the patient was discharged, following two segments of regression of sensory blockade. All patients were transferred to the post-anesthesia care unit (PACU) postoperatively, where they underwent observation at 10-minute intervals until discharge criteria were met.

Statistical analysis

Data were entered into Excel (Microsoft Corp., Redmond, USA) and analyzed using IBM SPSS Statistics software version 20 (IBM Corp., Armonk, USA). Descriptive statistics, including means, standard deviations, and percentages, were calculated for quantitative variables. Independent samples t-tests and chi-square tests were implemented to examine the study hypotheses. A p-value of < 0.05 was considered statistically significant.

Ethical consideration

Ethical committee approval was obtained from the Institutional Human Ethics Committee (IHEC) of Sree Balaji Medical College and Hospital, Chennai, prior to initiation of the study (reference no: 002/SBMCH/IHEC/2022/1872). Written informed consent was obtained from all participants after explaining the purpose, procedures, and voluntary nature of the study. Confidentiality of patient data was strictly maintained.

## Results

Overall, 88 participants were recruited for the study. Table 1 represents the socio-demographic characteristics of the participants. The current study, involving subjects receiving spinal anesthesia, revealed the following age distributions: in Group A, 14.8% were aged 20-30, 11.4% were 31-40, 13.6% were 41-50, and 10.2% were 51-60; Group B exhibited a similar breakdown, with 12.5% in the 20-30 age range, 13.6% in the 31-40 range, 12.5% in the 41-50 range, and 11.4% in the 51-60 range. Statistical analysis indicated no significant correlation between Group A and Group B with respect to age.

**Table 1:** Socio-demographic profile of the participants (N = 88) p < 0.05 indicates statistical significance. Chi-square test was applied.

Age-wise distribution				
Age group	Group A, n (%)	Group B, n (%)	Total, n (%)	p-value
20-30 years	13 (14.8)	11 (12.5)	24 (27.3)	0.9307
31-40 years	10 (11.4)	12 (13.6)	22 (25.0)	
41-50 years	12 (13.6)	11 (12.5)	23 (26.1)	
51-60 years	9 (10.2)	10 (11.4)	19 (21.6)	
Total	44 (50)	44 (50)	88 (100)	
Gender distribution				
Gender	Group A, n (%)	Group B, n (%)	Total, n (%)	p-value
Male	24 (27.3)	23 (26.1)	47 (53.4)	0.8308
Female	20 (22.7)	21 (23.9)	41 (46.6)	
Total	44 (50)	44 (50)	88 (100)	

In Group A, male patients comprised 27.3% of the study population, while female patients constituted 22.7%. Similarly, in Group B, 26.1% of the participants were male patients and 23.9% were female patients. Statistical analysis revealed no significant relationship between gender distribution.

Table 2 presents the distribution of heart rate among the study participants. Heart rate values were the highest at the start of the surgery (74.5 ± 5.4 and 73.3 ± 5.4 bpm) in both groups, with no statistical significance between the two groups.

**Table 2:** Comparison of heart rate between groups (N = 88) p < 0.05 indicates statistical significance. Independent samples t-test was applied.

Heart rate (bpm)	Group A, mean ± SD	Group B, mean ± SD	p-value
Baseline	73.3 ± 5.6	72.5 ± 4.5	0.765
At start of surgery	74.5 ± 5.4	73.3 ± 5.4	0.831
At end of surgery	72.5 ± 5.1	72.0 ± 5.6	0.946
At post-op ward 0 hours	72.8 ± 5.0	72.3 ± 4.5	0.937
At post-op ward 4 hours	73.3 ± 5.3	72.5 ± 5.6	0.634

Table 3 summarizes the distribution of systolic blood pressure among the study participants. Baseline systolic blood pressure (115.2 ± 6.6 and 114.0 ± 6.4 mmHg) was the highest in both groups, with no statistical significance between the two groups.

**Table 3:** Comparison of systolic blood pressure between groups (N = 88) p < 0.05 indicates statistical significance. Independent samples t-test was applied.

Systolic blood pressure (mmHg)	Group A, mean ± SD	Group B, mean ± SD	p-value
Baseline	115.2 ± 6.6	114.0 ± 6.4	0.745
At start of surgery	114.8 ± 6.1	113.3 ± 6.2	0.823
At end of surgery	113.3 ± 6.7	113.1 ± 6.4	0.936
At post-op ward 0 hours	113.5 ± 7.4	112.8 ± 4.5	0.736
At post-op ward 4 hours	111.8 ± 7.8	112.3 ± 6.6	0.481

Table 4 compares the diastolic blood pressure among the study population. Diastolic blood pressure values were the highest at the end of surgery for Group A (73.8 ± 6.5 mmHg) and at baseline for Group B (73.0 ± 6.7 mmHg), with no statistical significance noted.

**Table 4:** Comparison of diastolic blood pressure between groups (N = 88) p < 0.05 indicates statistical significance. Independent samples t-test was applied.

Diastolic blood pressure (mmHg)	Group A, mean ± SD	Group B, mean ± SD	p-value
Baseline	73.4 ± 6.3	73.0 ± 6.7	0.962
At start of surgery	72.6 ± 5.4	72.3 ± 5.4	0.971
At end of surgery	73.8 ± 6.5	72.5 ± 5.6	0.752
At post-op ward 0 hours	72.9 ± 5.6	72.3 ± 6.2	0.973
At post-op ward 4 hours	73.4 ± 6.4	72.0 ± 6.1	0.835

Table 5 demonstrates the distribution of SpO_2_ among the study participants. Oxygen saturation values were the lowest at the start of surgery for Group A (97.1 ± 1.3%) and after the first hour of surgery for Group B (97.1 ± 1.3%), with no statistical significance between the two groups.

**Table 5:** Comparison of peripheral oxygen saturation between groups (N = 88) p < 0.05 indicates statistical significance. Independent samples t-test was applied. SpO_2_: peripheral oxygen saturation

SpO_2 _(%)	Group A, mean ± SD	Group B, mean ± SD	p-value
Baseline	97.6 ± 0.9	97.6 ± 1.3	0.876
At start of surgery	97.1 ± 1.3	97.6 ± 1.4	0.983
At end of surgery	97.5 ± 1.1	97.2 ± 1.2	0.979
At post-op ward 0 hours	97.7 ± 1.5	97.1 ± 1.3	0.915
At post-op ward 4 hours	97.4 ± 1.3	97.6 ± 1.0	0.876

Table 6 shows the distribution of temperature among the study participants. Temperature values were the lowest four hours after surgery for both Group A (98.2 ± 0.3°C) and Group B (98.3 ± 0.4°C), with no statistical significance between the two groups.

**Table 6:** Comparison of temperature between groups (N = 88) p < 0.05 indicates statistical significance. Independent samples t-test was applied.

Temperature (℃)	Group A, mean ± SD	Group B, mean ± SD	p-value
Baseline	98.5 ± 0.4	98.5 ± 0.3	0.935
At start of surgery	98.6 ± 0.3	98.5 ± 0.4	0.911
At end of surgery	98.5 ± 0.3	98.5 ± 0.4	0.984
At post-op ward 0 hours	98.3 ± 0.4	98.4 ± 0.3	0.925
At post-op ward 4 hours	98.2 ± 0.3	98.3 ± 0.4	0.954

Table 7 presents the distribution of shivering grades across the two groups. The majority of participants in both groups did not develop shivering (grade 0). Higher grades of shivering (grades 2 and 3) were infrequent in both groups. Comparison using the Mann-Whitney U test demonstrated no statistically significant difference in shivering grades between Group A and Group B (U = 922.5, p = 0.39). This indicates that both magnesium sulfate doses produced comparable shivering outcomes.

**Table 7:** Comparison of shivering grades between groups (N = 88) p < 0.05 indicates statistical significance. Mann-Whitney U test was applied.

Grades of shivering	Group A, n (%)	Group B, n (%)	Total, n (%)	U-value	p-value
Grade 0 (Absent)	42 (47.7)	40 (45.5)	82 (93.2)	922.5	0.3900
Grade 1	1 (1.1)	1 (1.1)	2 (2.3)		
Grade 2	1 (1.1)	2 (2.3)	3 (3.4)		
Grade 3	0 (0.0)	1 (1.1)	1 (1.1)		
Grade 4	0 (0.0)	0 (0.0)	0 (0.0)		
Total	44 (50)	44 (50)	88 (100)		

## Discussion

The results of this study aligned with previous research. This study examined whether two routinely used IV magnesium sulfate doses (25 mg/kg and 50 mg/kg) differed in their ability to prevent shivering during spinal anesthesia. Magnesium has been shown to lower the shivering threshold by modulating NMDA receptor-mediated thermoregulatory pathways, which form the physiological rationale for evaluating these doses as highlighted by Wadhwa et al. [4]. The objective of this study was to compare these two dosing practices, which are already in routine departmental use, and to observe whether any meaningful differences emerged in shivering-related outcomes.

No statistically significant differences were detected between the two groups with respect to shivering incidence, shivering severity, or hemodynamic parameters. This aligns with findings from a study by Sahebanmaleki et al. [20], which also failed to demonstrate a clear dose-dependent advantage of magnesium within similar dose ranges. Given the low number of shivering events in our cohort, these results should be interpreted as “no difference detected” rather than implying true clinical equivalence between the two doses.

Variations between our findings and previous studies reporting stronger anti-shivering effects of magnesium may be explained by methodological differences. A study conducted by Gozdemir et al. used intrathecal routes or continuous core temperature monitoring, both of which may lead to more pronounced thermoregulatory effects than intermittent axillary measurement used in our study [21]. The lack of fine-resolution temperature monitoring in our protocol may therefore have limited the ability to detect early or subtle temperature-related changes associated with shivering.

The choice of 25 mg/kg and 50 mg/kg was based on existing practice patterns among anesthesiologists in the department and supported by clinical experience suggesting that magnesium within this range is safe and potentially beneficial, as highlighted by Low et al. [22]. Because these dose assignments occurred naturally without investigator intervention, the study preserved its observational design but simultaneously limited causal inference regarding any true dose-response relationship.

The absence of a control group (i.e., a group not receiving magnesium) further restricts interpretation. In a study conducted by Alipour et al. [23] evaluating magnesium against placebo reported clearer reductions in shivering, underscoring the importance of including a non-magnesium comparator when assessing efficacy. As such, the present findings describe only internal comparisons between two dosing routines and cannot be extrapolated to determine the absolute effectiveness of magnesium sulfate.

The very low incidence of shivering in both groups also limits interpretation. When event rates are low, statistical power is reduced, and subtle differences may remain undetectable. This limitation is consistent with a clinical study by Hashemi et al. [24] where small numbers of shivering episodes prevented firm conclusions about dose superiority or equivalence. Therefore, while both doses performed similarly in this cohort, the findings should not be interpreted as demonstrating therapeutic equivalence.

In addition, the short duration and relatively low thermal burden of the surgical procedures included in this study may have contributed to the minimal shivering observed. A study by Poovathai et al. [25] has shown that surgeries with limited exposure tend to produce fewer shivering events, reducing the likelihood of identifying dose-related differences even when they truly exist. This context dependence further highlights the challenge of evaluating anti-shivering interventions in low-risk populations.

Inter-individual differences in magnesium pharmacodynamics may also play a role. Variability in baseline magnesium stores, sympathetic tone, and vascular responsiveness can influence the thermoregulatory effects of magnesium, leading to heterogeneous clinical responses even within the same dose group, as highlighted by Mostafa et al. in their study [26]. This variability may have contributed to the comparable outcomes observed across dose regimens despite the difference in administered magnesium amounts.

Overall, this study contributes practice-based evidence indicating that no statistically significant difference was detected between the two magnesium sulfate dosing practices under the conditions evaluated. Future research with larger samples, inclusion of a non-magnesium control group, continuous core temperature monitoring, and assessment of higher-risk surgical populations is needed to more definitively determine whether dose-dependent effects exist and to identify an optimal dosing strategy for shivering prevention.

This study has several strengths, including its clinically relevant research question, the use of a simple and pragmatic comparative dosing framework reflecting real-world practice, application of a standard and validated shivering scale, and an adequate sample size for observational comparison. However, important limitations must be acknowledged. The non-randomized design introduces potential selection bias, and the absence of blinding may allow observer bias. As a single-centre study, external validity is limited. Although operating room temperature was maintained within a defined range, other potential confounders, such as fluid temperature and variation in surgical type or duration, were not strictly controlled. High-risk groups prone to shivering were excluded, subgroup analysis was not feasible due to the low event rate, and temperature was measured using axillary rather than continuous core monitoring. Additionally, the study lacked a pharmacokinetic justification for the chosen doses and did not include a control group, preventing conclusions regarding absolute efficacy. Together, these strengths and limitations should guide careful interpretation of the findings.

## Conclusions

This study found no statistically significant differences between the 25 mg/kg and 50 mg/kg magnesium sulfate dosing practices in terms of shivering incidence, shivering grades, temperature trends, or hemodynamic parameters. Although the 50 mg/kg group showed a numerically later onset of shivering, this difference was not statistically significant and should not be interpreted as a meaningful clinical effect.

Given the overall low incidence of shivering and the non-interventional observational design, no dose-related advantage could be established. The findings indicate that both dosing regimens performed similarly under routine clinical conditions, but do not allow conclusions regarding dose equivalence or therapeutic preference. Further randomized studies with larger sample sizes and an appropriate control group are required to more definitively assess the comparative effects of different magnesium sulfate doses on shivering prevention.
